# Dosimetric evaluation of synthetic CT image generated using a neural network for MR‐only brain radiotherapy

**DOI:** 10.1002/acm2.13176

**Published:** 2021-02-01

**Authors:** Bin Tang, Fan Wu, Yuchuan Fu, Xianliang Wang, Pei Wang, Lucia Clara Orlandini, Jie Li, Qing Hou

**Affiliations:** ^1^ Key Laboratory of Radiation Physics and Technology of the Ministry of Education Institute of Nuclear Science and Technology Sichuan University Chengdu Sichuan China; ^2^ Department of Radiation Oncology Radiation Oncology Key Laboratory Of Sichuan Province Sichuan Cancer Hospital & Institute Chengdu Sichuan China; ^3^ Department of Radiotherapy West China Hospital of Sichuan University Chengdu Sichuan China

**Keywords:** dosimetric comparison, generative adversarial network, image translation, MRI, synthetic CT

## Abstract

**Purpose and background:**

The magnetic resonance (MR)‐only radiotherapy workflow is urged by the increasing use of MR image for the identification and delineation of tumors, while a fast generation of synthetic computer tomography (sCT) image from MR image for dose calculation remains one of the key challenges to the workflow. This study aimed to develop a neural network to generate the sCT in brain site and evaluate the dosimetry accuracy.

**Materials and methods:**

A generative adversarial network (GAN) was developed to translate T1‐weighted MRI to sCT. First, the "U‐net" shaped encoder‐decoder network with some image translation‐specific modifications was trained to generate sCT, then the discriminator network was adversarially trained to distinguish between synthetic and real CT images. We enrolled 37 brain cancer patients acquiring both CT and MRI for treatment position simulation. Twenty‐seven pairs of 2D T1‐weighted MR images and rigidly registered CT image were used to train the GAN model, and the remaining 10 pairs were used to evaluate the model performance through the metric of mean absolute error. Furthermore, the clinical Volume Modulated Arc Therapy plan was calculated on both sCT and real CT, followed by gamma analysis and comparison of dose‐volume histogram.

**Results:**

On average, only 15 s were needed to generate one sCT from one T1‐weighted MRI. The mean absolute error between synthetic and real CT was 60.52 ± 13.32 Housefield Unit over 5‐fold cross validation. For dose distribution on sCT and CT, the average pass rates of gamma analysis using the 3%/3 mm and 2%/2 mm criteria were 99.76% and 97.25% over testing patients, respectively. For parameters of dose‐volume histogram for both target and organs at risk, no significant differences were found between both plans.

**Conclusion:**

The GAN model can generate synthetic CT from one single MRI sequence within seconds, and a state‐of‐art accuracy of CT number and dosimetry was achieved.

## 1 INTRODUCTION

Traditional radiotherapy workflow relies on computer tomography (CT) image for anatomy acquisition, tumors/organs delineation, patient positioning, and dose calculation. In the past two decades, magnetic resonance image (MRI) as the complementary modality to CT has been increasingly used in clinical routine as it can provide superior soft‐tissue contrast, especially for brain and pelvis site. Besides, the workflow in which CT images were replaced with MRI in each step of the entire radiotherapy chain, so‐called MR‐only workflow, is of growing interest. MR‐only workflow is reported to be advantageous, as it can avoid the registration error between CT and MRI, reduce inter‐ and intra‐observer contouring variation, lower the cost of radiotherapy, improve radiotherapy accuracy, reduce the patient exposure to ionization radiation,^1^, ^2^, ^3^, ^4^, ^5^, ^6^, ^7^, ^8^, ^9^, ^10^ etc.

The key challenge to MR‐only workflow is to extract the information of electron density from MRI for radiation dose calculation. Unlike CT number which can be directly converted to electron density, the pixel value in MRI only represents the magnetic relaxation time of tissue which has no direct correlation with electron density. However, the tissue relaxation time can be converted firstly into CT number and further into electron density, and the conversions can be categorized as three approaches.^11^ The first approach, in general, is to assign bulk densities for different tissues in MRI, which can be inaccurate and labor‐intensive because of manually contouring of tissue. The second approach is to establish CT number for the corresponding MRI voxel by aligning its voxel to an atlas with a pre‐known correlation between the MRI voxel location and the corresponding CT number. The third approach is the pixel‐wise conversion, which establishes a correlation between pixel values of MRI and CT by training through machine learning. Among those approaches, neural networks as a specific method of machine learning stands out for its advantage of high accuracy and automation, and it is considered as the potential priority method for clinical MRI‐only radiotherapy workflow.

Deep convolutional neural network (DCNN) has been reported successful in a wide range of medical applications. Several studies utilized the convolutional neural network to perform the synthesis of CT from a variety of MRI sequences. Han^12^ and Liu^13^ applied the u‐net^14^ based network to convert MRI to sCT pixel by pixel. The encoder‐decoder architecture in their networks enable the learning of a hierarchy of features from MRI through a downsampling process, then those features in various resolution were combined to generate high‐resolution CT image through an upsampling process. Besides, the generative adversarial network (GAN) tailored for image‐to‐image translation has been applied in the translation of MRI to CT.^15^, ^16^, ^17^, ^18^, ^19^ Those U‐net based networks contain only the generator of CT image, while the GAN contain an additional adversarial network as the discriminator which would compete with the generator to distinguish generated CT images from real CT. Although those deep learning‐based methods mentioned above have achieved state‐of‐the‐art performance, there still a lot of factors, that is, MRI sequence, registration method, loss function, worthy spending efforts on since many of them can be influential to the results. In this study, we aimed to develop a GAN model to translate clinical standard MRI to synthetic CT, and evaluate its accuracy in terms of image pixel value and clinical radiotherapy dosimetry.

## 2 MATERIALS AND METHODS

### 2.A Patient data collection

Thirty‐seven brain cancer patients who had undergone external radiotherapy from July 2019 to April 2020 in our department were enrolled. Their median age is 50.4 (range: 15 ~ 83). For each patient, the MR image was acquired on a 3T scanner (Siemens, Erlangen, Germany) with the following parameters: T1 TIRM Dark Fluid spin echo sequence, 18 ms echo time, 2000 ms repetition time, 0.718 × 0.718 mm transversal voxel dimensions, 6.5 mm slice thickness, 896.4 ms inversion time, 150°flip angle. On the same day, the CT image was acquired on a CT scanner (Philips Healthcare, Eindhoven, The Netherlands) with 120 kV and 300 mA tube current, 0.625 × 0.625 mm transversal voxel dimensions, 3 mm slice thickness. All MRIs were rigidly registered to the corresponding CT based on mutual information (MI) and resampled to the same voxel size as CT images.

### 2.B Generative adversarial network

**FIG. 1:**
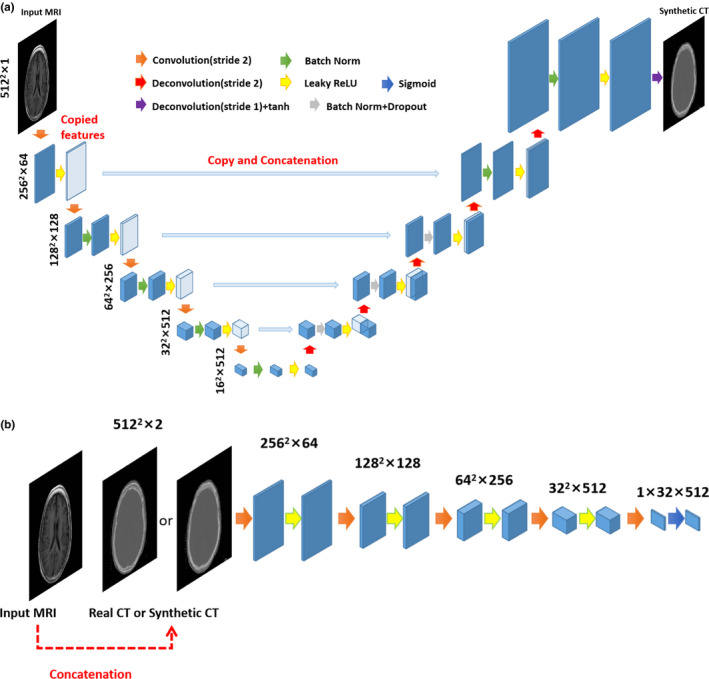
Architecture of generator (a) and discriminator (b) of the generative adversarial network. The blue cubes represent the feature maps extracted by the convolutional and deconvolutional layers; the arrows of different colors denote different operations; the x‐y‐z size is noted for each row (a) and block (b) of feature maps; white cubes represent copied feature maps.

A conditional generative adversarial network similar to “pix2pix” was adopted here. Two networks namely generator and discriminator comprised of the network. The paired MRI and CT images of each patient were feed into the generator for learning the mapping from CT from MRI, so that the generator can generate sCT from an input MRI. Then the discriminator was trained to compete with the generator and distinguish sCT from the corresponding real CT as well as possible. Through the training of generator and adversarially training of discriminator, the network would converge to its best performance. The detailed architectures of generator and discriminator were illustrated in Figs. 1(a) and 1(b), respectively.

We adopted a “U‐net” shaped encoder‐decoder network as the generator. For the encoder, we have five convolutional layers with a filter size 4 × 4 and a stride of 2 to downsample the input 2D MRI slices from size 512 × 512 to 16 × 16. Each convolutional layer was followed by batch normalization and a Leaky rectified linear unit (Leaky ReLU). For the decoder, a mirrored upsampling process with skip connection to corresponding encoder layers decodes the low‐resolution feature maps into 2D synthetic CT. The features from each encoder layer were copied and concatenated with the corresponding feature before each deconvolution layer except the first and last one. The dropout layers were applied after the first three batch normalizations in the decoder network to improve network generalization.^20^ Compared to the original U‐Net, the total number of convolutional layers was reduced from 19 to 11. Another modification to U‐Net is that all pooling layers and unpooling layers were replaced by convolutional and deconvolutional layers, because fractionally strided convolutional layers can be trained to produce dense high‐resolution feature maps, while unpooling layers use memorized pooling indices from maxpooling layers to produce sparse high‐resolution feature maps.^21^

The discriminator network consisted of five convolutional layers with a filter size 4x4 and a stride of 2. The concatenation of input MRI and synthetic or real CT was feed to the first convolutional layer. The leaky ReLU followed each convolutional layer except the last one, which was followed by a sigmoid function then output a score map of shape 1 × 32 × 512 to distinguish between synthetic CT and real CT.

The loss function used in the generator network was mean absolute error (MAE) as defined in Section 2.D, to represent the pixel‐wise difference between synthetic CT and real CT. For discriminator, we adopt the least square loss function since it strongly penalized the fake samples away from decision boundary and improve the stability of learning process.^22^ The Loss term can be expressed as follows:

$$\log\left(D{\left(x\right)}^{2}\right)+\log\left({\left(1‐D\left(G\left(y\right)\right)\right)}^{2}\right)$$

where the D and G represented the discriminator and generator, respectively, and x,y represented the pair of real CT and MRI, G(y) is output of generator, namely the synthetic CT.

### 2.C Training and cross validation

All 48 patients were randomly divided into training group (27 patients) and testing group (10 patients), and the training group were further split into five folds for cross validation. In each run, four folds were used to train the model and the remaining one fold was used to validate the model performance, till all folds were used for validation. Each trained model was used to generate synthetic CT for all patients in testing group.

The network weights were initialized using Xavier^23^ and updated using the ADAM algorithm^24^ with a fixed learning rate of 0.0002. The batch size was set to 20 to make best use of video memory, and around 32000 steps (720 epochs) were taken to converge each training. The training was performed on a 64‐bit Windows workstation, with an Intel Core i7 CPU and an NVIDIA GeForce GTX Titian X graphics card with 12 G RAM.

### 2.D Evaluation of synthetic CT

For each testing patient, the mean absolute error (MAE) of each pixel value within patient body contour between sCT and real CT was calculated as follows:

$$\text{MAE}=\frac{\sum_{i=1}^{n}\left|I_{\mathrm{CT}}\left(i\right)‐I_{\mathrm{sCT}}\left(i\right)\right|}{N}$$

The peak signal to noise ratio (PSNR) is also evaluated as follows:

$$\text{PSNR}=20\log_{10}\frac{\mathrm{MAX}}{\mathrm{MSE}}$$

where MAX strands for maximum signal value of real CT, and MSE stands for mean square error calculated by

$$\text{MSE}=\frac{\sum_{i=1}^{n}{\left(I_{\mathrm{CT}}\left(i\right)‐I_{\mathrm{sCT}}\left(i\right)\right)}^{2}}{N}$$

### 2.E Treatment planning and dose calculation

The clinical plans with dual Volumetric Modulated Arcs Therapy (VMAT) delivery technique were optimized on real CT (planning CT) for 10 patients in testing group by the Eclipse 11.0 treatment planning system (Varian Medical System, Palo Alto, USA). For each patient, the synthetic CT was imported into Eclipse, and rigidly registered with real CT. Then all the contours of target and organs at risk were transferred from real CT to sCT. The clinical VMAT plan was also transferred based on the registration of images. The same table of Electron density to Housefield Unit was applied to both CT, then the dose distribution on both sCT and CT were calculated by Anisotropic Analytical Algorithm (AAA) with a dose matrix resolution of 0.3 × 0.3 × 0.3 mm^3^.

The Dose‐Volume Histograms (DVHs) for both plans of all testing patients were analyzed. Moreover the gamma analysis was performed between the dose distributions on real and synthetic CT at 3%/3 mm and 2%/2 mm criteria, respectively.

## 3 RESULT

Each training of model parameters with our dataset cost around 15 h, while generating a single synthetic CT from an input MRI using the trained model took only 15 s on average.

### 3.A Image comparison between synthetic and real CT

**FIG. 2:**
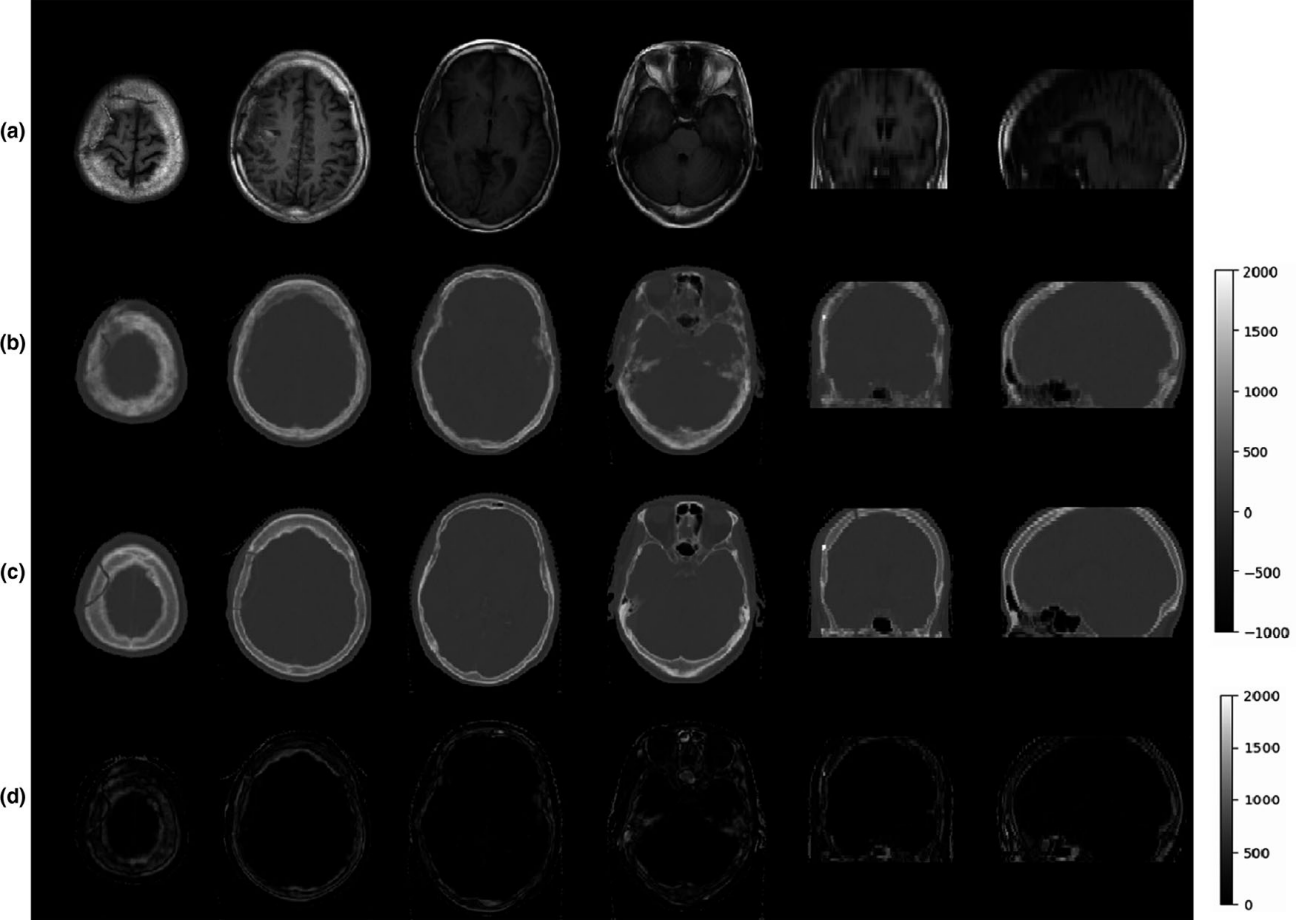
Comparison of (a) T1‐weighted TIRM Dark Fluid MRI, (b) synthetic computed tomography (CT), (c) planning CT and (d) difference map in transverse, sagittal, and coronal views for one example patient.

For all testing patients, the average and standard deviation of MAE between synthetic and real CT were 60.13 ± 13.72 HU, 60.51 ± 14.23 HU, 61.14 ± 12.56 HU, 62.28 ± 13.85 HU, 59.38 ± 13.23 HU using training folds 1, 2, 3, 4, and 5, respectively. The average of MAE and PSNR over all cross validation dataset was 60.52 ± 13.32 HU and 49.23 ± 1.92 dB. Figure 2 showed the comparison of MRI, synthetic CT, planning CT and difference map for one example patient. A good visual result of CT synthesis by GAN was shown, except for some blurry area in the vicinity of the interface between skull and brain tissue.

### 3.B Dosimetric comparison between synthetic and real CT

**FIG. 3:**
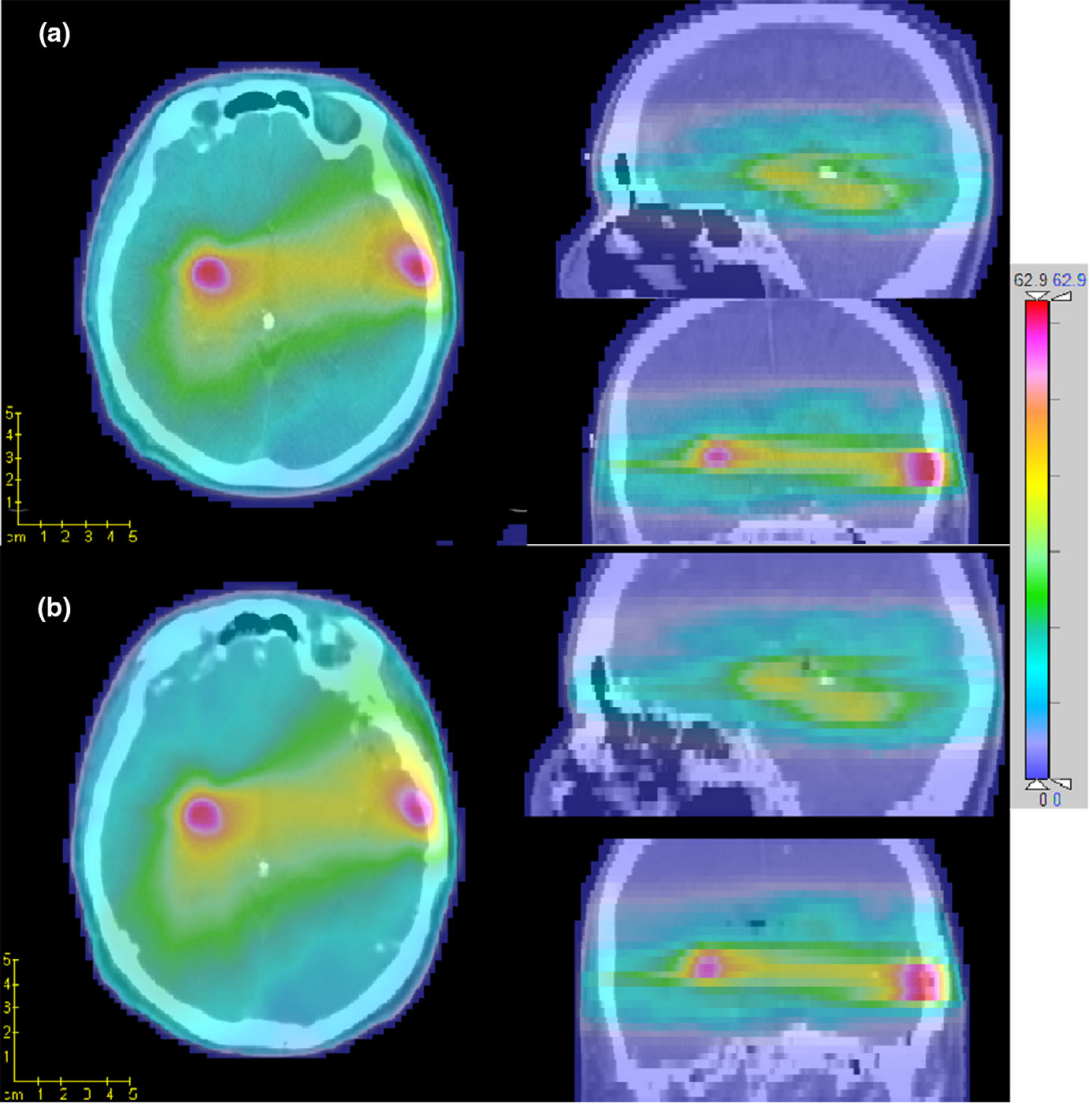
Dose distribution of a clinical VMAT plan calculated on planning (a) and synthetic (b) computed tomography.

**FIG. 4:**
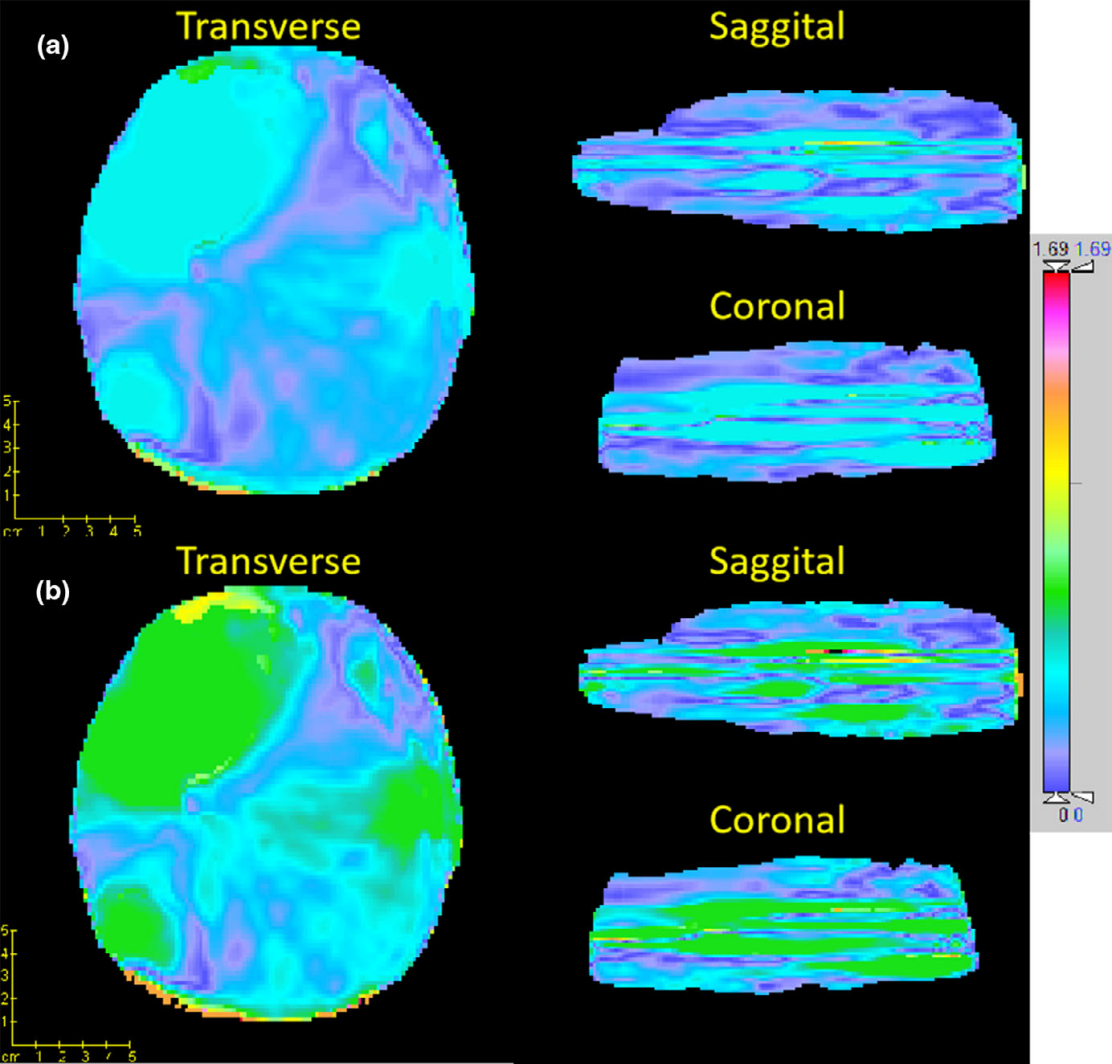
Gamma analysis between the dose distribution of synthetic and planning computed tomography in transverse, sagittal, and coronal planes with criteria of 3 mm/3% (a) and 2 mm/2% (b), respectively.

**FIG. 5:**
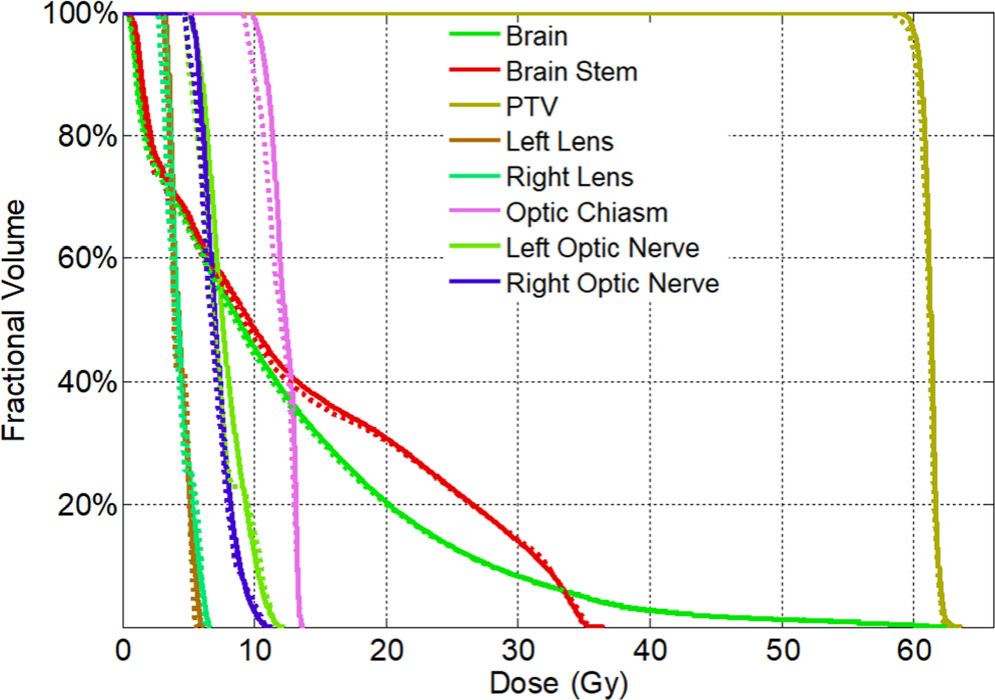
Comparison of DVH of a clinical VMAT plan calculated on the synthetic computed tomography (CT) (Dashed) and planning CT (Solid).

For each testing patient, a VMAT radiotherapy plan was optimized on the planning CT then calculated again on the corresponding sCT. For the comparison of dose distribution, the result was illustrated in Fig. 3 for a representative patient, which showed a very similar distribution on both CTs. The gamma analysis was also applied over all testing patients and the mean value of 99.76% (range 99.31% to 100%) and 97.25% (range 95.95% to 99.65%) were obtained with criteria of 3 mm/3% and 2 mm/2%, respectively. The main dosimetric discrepancy (gamma value > 1) located at the skin surface where the photon beam entered, also at the dose falling region around the tumor, as shown in Fig. 4 for one example patient. Besides, The DVH parameters of target and organs at risk (OARs) on both CTs were calculated with the contours delineated on planning CT, then compared with nonparametric paired‐sample Wilcoxon signed‐rank sum test, the average of each parameters were listed in Table 1. No significant differences were found for both target and OARs. The comparison of DVH for one example patient was illustrated in Fig. 5.

**TABLE 1:** Comparison of mean DVH parameters of plans on synthetic and planning computed tomography (CT) for 10 testing patients

DVH parameter		Synthetic CT (range)	Planning CT (range)	Dose difference	Wilcoxon *P*‐value
PTV	D95 (Gy)	60.17 (59.92 to 60.79)	60.15 (59.86 to 60.95)	0.033%	0.623
	Dmean (Gy)	60.95 (60.55 to 62.22)	61.03 (60.65 to 62.25)	−0.13%	0.16
	Dmax (Gy)	63.19 (62.43 to 64.43)	63.27 (62.5 to 64.66)	−0.13%	0.159
Len	Dmax (Gy)	3.03 (0.79 to 6.71)	2.99 (0.8 to 6.44)	1.33%	0.888
Brain stem	D1cc (Gy)	18.37 (3.07 to 34.1)	18.34 (3.11 to 33.9)	0.16%	0.499
Optic nerve	Dmax (Gy)	6.13 (1.38 to 12.27)	6.17 (1.3 to 12.6)	−0.65%	0.091
Optic chiasma	Dmax (Gy)	7.76 (1.25 to 13.59)	7.82 (1.21 to 13.85)	−0.77%	0.26
Brain	Dmean (Gy)	22.27 (15.23 to 31.75)	21.99 (15.1 to 31.4)	1.27%	0.141

On the converse, a VMAT plan was optimized on synthetic CT following clinical protocols, then transferred and calculated on planning CT. and the gamma analysis showed 99.96% and 97.99% with criteria of 3 mm/3% and 2 mm/2%, respectively, which is close to the comparison result when the VMAT plan was optimized on the planning CT as stated before.

## 4 DISCUSSION

To explore the feasibility of MR‐only radiotherapy, we developed a generative adversarial network to generate synthetic CT from one single MRI sequence. Through the training by pairs of MRI and CT of brain scanning, the GAN model could translate the input T1‐Weighted TIRM Dark Fluid MRI into CT images. In the field of translating MRI to CT, most of previous studies required dedicated MRI sequences (dUTE and Dixon), or a combination of multiple MRI modalities as the input data to allow a better contrast of bone, even some manually contouring coincide with them.^11^ Considering keeping acquisition time as short as possible and avoiding labor‐intensive processing, the direct utilizing of one single clinically common sequence is favorable in the workflow of translating MRI to CT, which motivated us to use T1‐weighted clinical sequence in this study.

The average of MAE between synthetic CT and planning CT was 60.77 ± 13.99 HU, which is better than some previously reported results using atlas‐based or machine learning methods.^25^, ^26^, ^27^, ^28^, ^29^, ^30^, ^31^ A few studies used the neural network,^12^, ^13^, ^16^, ^17^, ^32^ either GAN or CNN. Although different network architectures and modalities of training data, finally we achieved similar or even better results compared to theirs.

The field of MRI translation to CT covers both diagnostic and therapeutic radiology. Since our prior goal was to use the synthetic CT from MRI for dose calculation in clinical workflow, the dosimetric performance of synthetic CT was also evaluated by comparing dose distribution of the same clinical plan delivered, respectively, on synthetic CT and planning CT. A mean pass rate of 99.79% with 3 mm/3 % criterion and 97.23% with 2 mm/2% criterion was similar to other studies using deep learning approaches.^13^, ^33^ While Kazemifar^17^ also utilized GAN network and show a slightly higher pass rate of 98.7% with 2 mm/2%. One of the distinctions in their study was the use of mutual information as the loss function, instead of MAE in ours. Since the performance of the neural network was also influenced by other factors, i.e. training data, the improvement by using MI still needs more studies to verify.

For the evaluation of DVH, we choose D95, mean dose and maximum dose for PTV, and D1cc, mean dose and maximum dose for OAR as pointed by the reference,^34^ which are highly relevant clinical information. And all the metric of PTV and OAR showed no significant difference through Wilcoxon signed rank‐sum test, which suggests a minimal risk from the use of sCT for dose calculation.

Furthermore, to explore the feasibility of plan optimization on synthetic CT, we followed clinical protocols and optimized beam fluences on synthetic CT for one patient, and transferred it to corresponding CT image for dose calculation. The pass rate of gamma analysis between dose distributions did not show significant difference referring to the comparison of dose recalculation of plan optimized on planning CT. This indicates that the direct utilizing of the sCT on beam fluences optimization does not bias plans on dosimetric perspective.

Calculation on synthetic CT generated from MRI has gained growing interest, mainly due to the enthusiasm of developing MR‐only workflow. On one hand, the advent of MR‐guided Linac^35^ and MR‐guided ^60^Co radiotherapy^36^ encouraged the exploration of MR‐guided adaptive radiotherapy workflow, while the MR‐only workflow seems to be a promising approach that has the potential minimize the cost of the daily adaptive routine. On the other hand, even though the majority of clinic centers might not be equipped with MRI‐guided devices; however, the MRI‐simulators or MRI diagnostic scanners would likely be present in near future, so they have the chance to benefit from MR‐only workflow. Thus, a fast and accurate approach of translating MRI to CT is desirable to be implemented in the clinical workflow. Our studies show that the translation by neural network required only took around ten seconds on one single GPU, and a state‐of‐the‐art dosimetric accuracy can be achieved for brain site.

## 5 CONCLUSION

The GAN model can perform a fast synthesis of CT from a single clinical MRI sequence, and high accuracy of CT number and dosimetry was achieved. This accomplishment allows the elimination of CT scanning in the radiotherapy workflow when CT images would not be helpful for tumor delineation since MRI can provide better resolution of soft tissues.

## CONFLICT OF INTEREST

The authors have no relevant conflict of interest to disclose.
